# Glucosuria as an early marker of late-onset sepsis in preterms: a prospective cohort study

**DOI:** 10.1186/s12887-015-0425-5

**Published:** 2015-09-17

**Authors:** Jolita Bekhof, Boudewijn J. Kollen, Joke H. Kok, Henrica L. M. Van Straaten

**Affiliations:** Princess Amalia Children’s Clinic, Isala, Dr van Heesweg 2, PO Box 10400, 8000 GK Zwolle, The Netherlands; Department of General Practice, University MedicalCenter Groningen, University of Groningen, Groningen, The Netherlands; Department of Neonatology, Academic Medical Center, Amsterdam, The Netherlands

**Keywords:** Premature infant, Infection, Nosocomial sepsis, Signs and symptoms, Diagnosis, Glucose, Hyperglycaemia

## Abstract

**Background:**

Early and accurate diagnosis of late-onset sepsis (LONS) in preterm infants is difficult since presenting signs are subtle and non-specific. Because neonatal sepsis may be accompanied by glucose intolerance and glucosuria, we hypothesized that glucosuria may be associated with LONS in preterms, in an early stage. We aim to evaluate the association of glucosuria and late-onset neonatal sepsis (LONS) in preterm infants, in an attempt to improve early and accurate diagnosis of LONS.

**Methods:**

We performed a prospective observational cohort study in 316 preterms (<34 weeks). We daily measured glucosuria and followed patients for occurrence of LONS, defined as clinical and blood culture-proven sepsis occurring after 72 h. Attending physicians were blinded to glucosuria results. We assessed the diagnostic value of glucosuria for clinical and blood culture-proven LONS using logistic regression analysis.

**Results:**

Glucosuria was found in 65.8 % of 316 preterm patients, and sepsis was suspected 157 times in 123 patients. LONS was found in 47.1 % of 157 suspected episodes. The presence of glucosuria was associated with LONS (OR 2.59, 95 % CI 1.24–5.43, *p* = 0.012) with sensitivity 69.0 % and specificity 53.8 % (Likelihoodratio 1.49). After adjustment for gestational age, birth weight, and postnatal age, this association weakened and was no longer significant (adjusted OR 2.16; 95 % CI 0.99–1.85, *p* = 0.055). An increase in glucosuria 48–24 h before onset of symptoms was not associated with LONS.

**Conclusion:**

In preterms glucosuria is associated with LONS within 24 h, however this association is too weak to be of diagnostic value.

## Background

Late-onset neonatal sepsis (LONS), mostly defined as neonatal sepsis occurring after three days of age, is an important cause of morbidity and mortality in preterm infants [1]. Early and correct diagnosis of LONS in preterm infants is challenging because presenting signs are subtle and non-specific [2]. Several screening tools have been investigated to improve early diagnosis of LONS, such as a combination of clinical signs [3–6], haematological biomarkers [5–9], changes in microcirculation [10], heart rate monitoring [11, 12] and use of central-peripheral temperature gradient [13].

Disturbed glucose homeostasis is frequently seen in prematurely neonates. Hyperglycaemia has been found in 25 to 80 % of premature newborns, depending on their gestational age and birth weight [14–16]. Because of inadequate hepatic and diminished pancreatic insulin secretory responsiveness, the risk of hyperglycaemia increases in stressful episodes, such as sepsis [17, 18]*.* Together with a lower renal threshold, such glycaemic instability may produce glucosuria in preterm infants, even in absence of hyperglycemia [19].

Although it has been recognized that hyperglycaemia may be an important early sign in neonatal sepsis [18, 20, 21], monitoring of blood glucose requires repeated blood sampling, with its disadvantages of discomfort, stress and the risk of iatrogenic anemia and blood transfusions. To the best of our knowledge, the usefulness of non-invasive measurement of glucosuria as an early sign of neonatal sepsis has not been prospectively investigated.

Therefore, the aim of this study was to evaluate the diagnostic value of changes in glucosuria in the early detection of LONS in preterm infants.

## Methods

### Patients

This study was part of a prospective cohort study investigating clinical signs and symptoms in preterm infants with suspected LONS, undertaken from July 2005 until November 2007, at our level-III neonatal intensive care unit (NICU) in Zwolle, the Netherlands [6]. Eligible patients included all patients with a postconceptional age < 34 weeks, > 72 h after birth who had not been on antibiotic therapy for the last 24 h. Patients were followed until a corrected gestational age of 35 weeks or until discharge to other hospitals before 35 weeks [22].

### Glucosuria measurement

We prospectively collected data on glucosuria and the occurrence of late-onset sepsis on a daily basis. Combur reagent strips (Combur^3^Test® Roche) were used to test urine samples for glucosuria, using a semi-quantitative scoring system of 0 (no glucosuria), 1+ (urine glucose level 0.6–5.0 mmol/L), 2+ (3.3–7.7 mmol/L), 3+ (14.8–19.2 mmol/L) and 4+ (52.8–57.2 mmol/L), according to the product information. Reagent strips were pressed into the wetted diaper directly or applied to urine collected by needle and syringe from a piece of gauze in the diaper. Glucosuria was assessed at least 8 times each day of admission in each infant until a corrected age of 35 weeks. Measurement of glucosuria was performed by the attending nurse and were collected only for research purposes. Treating clinicians were unaware of glucosuria results.

### Definitions

A change in glucosuria was defined as an increase of at least one category; category 1+ and 2++ were pooled since we previously showed that these categories cannot be reliably distinguished, because the glucosuria ranges in these two categories overlap [22, 23].

An episode of suspected sepsis was defined as a clinical suspicion by the attending neonatologist. At the onset of each episode, data on clinical signs were assessed in a standardised way, i.e. the physician completed a form with clinical signs on which the suspicion of LONS was based. In addition, routine laboratory investigations, including blood cultures, C-reactive protein (CRP) and full blood count were performed [6]. We only included the first episode of suspected LONS.

The outcome of each episode was classified in 3 mutually exclusive categories: blood-culture proven sepsis, clinical (blood-culture negative) sepsis and rejected sepsis. The classification was made by the researchers based on the course of the episode after the start of antibiotics, using the following predefined definitions of LONS. *Blood-culture proven sepsis* was defined as an episode with positive non-contaminated blood-culture. A positive blood-culture with organisms regarded as commensals (predominantly Coagulase-negative Staphylococcus) was defined as contamination. However a positive blood culture with skin commensals was defined as proven sepsis when the same organism was found in at least two blood cultures and/or signs of catheter-related sepsis were present, such as inflammation of the skin at the site of line insertion. *Clinical sepsis* was defined as strong clinical suspicion of sepsis as defined by the attending neonatologist or raised CRP during the first 3 days after the onset of suspected infection (>10 mg/l) or positive haematological markers (leukopenia ≤ 5 x 10^9^/l, leucocytosis ≥ 25 x 10^9^/l, or left shift of the differential count), despite a negative blood culture. *Rejected sepsis* was defined as an episode with negative blood culture (plus CRP <10 mg/l and negative haematological markers) or an episode in which no blood culture was performed and no antibiotics were started. In all our analyses and results, LONS was defined as *blood-culture proven and/or clinical sepsis*, unless stated otherwise.

### Statistical analysis

Associations of the degree of glucosuria with patient characteristics and LONS were analysed by Chi^2^.

Association between the presence of glucosuria as well as an increase in glucosuria and the occurrence of LONS was analysed in the group of patients with suspected infection using multivariate logistic regression analysis, adjusting for birth weight, gestational and postnatal age, and the known risk factors for glucosuria. Furthermore, to determine whether glucosuria has an added value to diagnose LONS, a multivariable model was developed in which the association between LONS and glucosuria was estimated with and without correction of some major clinical signs in this study population, as we reported earlier [6], i.e. increased respiratory support, lengthened capillary refill, grey skin and the presence or recent removal of a central venous catheter.

Analyses were performed using SPSS version 20.0.

### Consent and ethical approval

The study was approved by the local medical ethical committee of our hospital (METC Isala Zwolle, The Netherlands: Isalakl075). Written informed consent was obtained from the parents. We state that we have complied with the World Medical Association Declaration of Helsinki regarding this study.

## Results

During the 2-years study period, a total of 316 of 360 eligible patients < 34 weeks were included. Reasons for exclusion were: short admission < 1 day (*n* = 7), antibiotics during the entire admission (*n* = 31) or missing measurements of glucosuria (*n* = 6). A total of 187 episodes of suspected sepsis occurred in 142 patients. To avoid bias due to double counting of patients who experienced more than one episode of suspected sepsis, we only included the first episode of suspected sepsis in our analyses. After excluding 30 of 187 episodes, because of missing glucosuria measurements, we found 157 episodes of suspected infection in 123 patients, resulting in a total inclusion of 123 sepsis episodes. Patient characteristics and their suspected sepsis episodes are presented in Table 1.

**Table 1:** Characteristics of the study population and suspected sepsis episodes

	Patients with suspected infection
	*n* = 123
Gestational age, weeks^+days^	29^+1^ (2^+1^)
Birthweight, g	1187 (345)
Male sex, n (%)	70 (56.9 %)
Died during admission, n (%)	1 (0.8 %)
Age at onset of suspected infection, days	12 (7–18)
Follow-up, days	24 (14–35)
Respiratory symptoms	
(Increase in) apnea, bradycardia and/or cyanotic spells	56 (45.5 %)
Increased respiratory support^a^	53 (43.1 %)
Circulatory symptoms	
Pallor/gray skin	61 (49.6 %)
Capillary refill time > 2 s	49 (39.8 %)
Tachycardia	44 (35.8 %)
General symptoms	
Temperature instability	86 (69.9 %)
Lethargy	56 (45.5 %)
Irritability	14 (11.4 %)
Laboratory values	
C-Reactive protein (mg/l)	2 (0–10)
Leucocytes (10^E^9/l)	14.0 (8.9–18.0)
Risk factors	
CVC in last 24 h	39 (31.7 %)
Mechanical ventilation	12 (9.8 %)

Mean (standard deviation) for gestational age, birth weight. For age at onset and follow-up in days median (p25 and p75) are given because of skewed distribution

Data from patients who experienced more than one episode of suspected infection are from their first episode. For characteristics of sepsis episodes numbers (percentage) are given, except for “Laboratory values” where median (p25-p75) are presented. CVC central venous catheter

^a^Increased in respiratory support: intensifying the modus, i.e. low flow, CPAP or endotracheal ventilation and/or degree of respiratory support)

### Glucosuria

Glucosuria within the individual patient at any time during the study period and in varying degrees, was found in the majority (208/316, 65.8 %) of patients (+ or ++ in 127/316 (40.2 %), +++ in 56/316 (17.7 %), and ++++ in 25/316 (7.9 %)). Glucosuria was negatively associated with gestational age (OR_per week gestational age_ 0.64; 95%CI 0.56–0.73, *p* < 0.001) and birth weight (OR_per 100g birth weight_ 0.80; 95%CI 0.75–0.85, *p* < 0.001). Glucosuria was found in 130 (92.2 %) of 141 patients with gestational age ≤ 30 weeks, as compared to 64 (57.7 %) in 111 patients with gestational age 31–32 weeks and 14 (21.9 %) in 64 patients with gestational age 33–34 weeks (*p* = 0.001). Glucosuria was more common in patients with birth weight < 1200 g (99/109, 90.8 %) than in those ≥ 1200 g (109/207, 52.7 %, *p* < 0.001).

Glucosuria was equally common in males and females (115/181, 63.5 % versus 93/135, 68.8 %; *p* = 0.321).

### Sepsis diagnosis

A diagnosis of LONS was made in 58 (47.2 %) of the 123 suspected sepsis episodes: 21 (17.1 %) clinical sepsis and 37 (30.1 %) culture-proven sepsis. In the culture proven sepsis episodes the following microbacteriae were found: Staphylococcus Epidermidis (21.6 %), Staphylococcus Aureus (10.8 %), other Staphylococcus (37,8 %) Bacillus Cereus (18.9 %), gram negative strains (5.4 %), Streptococcus (2.7 %) and Candida (2.7 %).

### Association of glucosuria with late onset sepsis

#### Absence of glucosuria

The 108 patients who never had glucosuria had fewer suspected sepsis episodes (13.9 %) than the 208 patients with glucosuria ever (60.1 %, OR 9.34; 95 % CI 5.06–17.22, *p* < 0.001). This association remained significant after correction for gestational age and birth weight (OR 4.19; 95 % CI 2.11–8.32, *p* < 0.001).

#### Glucosuria in the period before onset of LONS

Suspected sepsis episodes were commonly preceded by glucosuria between 48 and 24 h (55.7 %), or in the 24 h before onset of clinical suspicion (56.9 % of episodes). Glucosuria 48–24 h before onset was not associated with confirmed LONS, defined as clinical ánd proven sepsis (OR 1.06, 95 % CI 0.52–2.15, p 0.883), nor with solely culture-proven sepsis (OR 0.98, 95 % CI 0.45–2.12, *p* = 0.951). The presence of glucosuria in the 24 h before clinical suspicion was positively associated with LONS (OR 2.59, 95 % CI 1.24–5.43, *p* = 0.012), but not with culture-proven sepsis (OR 1.61; 95 % CI 0.72–3.56, *p* = 0.244, Table 2). After adjustment for gestational age, birth weight and postnatal age, the association between the degree of glucosuria in the 24-h before the episode and confirmed LONS became non-significant (OR 2.16, 95 % CI 0.97–4.84, *p* = 0.060). Diagnostic value of the presence of glucosuria in the 24-h period before suspected LONS is presented in Table 3.

**Table 2:** Association of glucosuria in the 24-h period before clinical suspicion of late-onset neonatal sepsis

*n* = 123		Prevalence	Rejected infection	Clinical sepsis	Proven sepsis (positive bloodculture)
			*n* = 65	*n* = 21	*n* = 37
Maximum glucosuria 24 h before onset of suspected infection	0	53 (43.1 %)	35 (66.0 %)	5 (9.4 %)	13 (24.5 %)
	+/++	54 (43.9 %)	23 (42.6 %)	12 (22.2 %)	19 (35.2 %)
	+++	12 (9.8 %)	6 (50.0 %)	1 (8.3 %)	5 (41.7 %)
	++++	4 (3.2 %)	1 (25.0 %)	3 (75.0 %)	0 (0 %)
Increase in glucosuria		28 (22.8 %)	11 (39.0 %)	6 (17.1 %)	11 (30.1 %)

**Table 3:** Diagnostic value of glucosuria 0–24 h before clinical suspicion of LONS in preterm infants

123 episodes of suspected LONS		Confirmed clinical and culture proven LONS					
		*n* = 58 (47.2 %)					
	prevalence	sensitivity	specificity	ppv	npv	LR^+^	LR^−^
Presence of glucosuria	56.7 %	69.0 %	53.8 %	57.1 %	66.0 %	1.49	0.58
Increase in glucosuria	22.8 %	29.3 %	83.1 %	60.7 %	56.8 %	1.73	0.85

*LONS* Late-onset neonatal sepsis, *ppv* positive predictive value, *npv* negative predictive value, *LR* Likelihoodratio

#### Increase in glucosuria in the period before onset of LONS

A change in glucosuria in the 24-h preceding before the onset of suspected LONS was found in 28 (22.8 %) of episodes (decrease in 9.7 %, no change in 67.5 %, one category increase in 20.3 %, 2 categories increase in 2.4 % (Table 2)).

An increase in glucosuria in the 24 h before onset of symptoms was not associated with confirmed clinical and culture-proven LONS (OR 2.04, 95 % CI 0.86–4.81) nor for solely culture-proven sepsis (OR 1.26 95 %-CI 0.71–4.15, *p* = 0.230). Diagnostic value of the increase in glucosuria in the 24-h period before suspected LONS is presented in Table 3.

#### Comparison of glucosuria with other clinical signs and symptoms in LONS

Correction with some major clinical signs that were shown to be the strongest predictors of LONS in this study population, as we reported earlier [6], weakened the presence of glucosuria to non-significant (OR 1.6; 95 % CI 0.61–4.00, p 0.356). When fitting all the sign variables in a multivariable model, using backward logistic regression analysis, the glucosuria as well as an increase in glucosuria were left out of the final model, meaning that glucosuria does not add to the predictive value of the combination of the other signs and symptoms.

## Discussion

### Principal findings

This study shows that glucosuria is very common in preterm infants, with increasing prevalence with lower gestational age, lower birth weight, and with the occurrence of late onset sepsis. Although glucosuria in the 24 h before clinical suspicion of sepsis is associated with confirmed LONS, the diagnostic value is only marginal. More-over glucosuria was not associated with culture proven sepsis. This is probably due to the fact that glucosuria is merely associated with gestational age and weight, which are well-known, and stronger risk factors for the occurrence of LONS. The measurement of glucosuria doesn’t seem to be of added value to detecting LONS, when compared to the stronger clinical signs, such as increased respiratory support severe respiratory or circulatory symptoms [6]. Because glucosuria was more strongly associated with confirmed LONS (either clinical sepsis or culture-proven sepsis) than with solely culture-proven LONS, it appears that (an increase in) glucosuria can possibly be regarded as a sign of stress and not as a specific marker of infectious sepsis. This is in accordance with earlier reports of the association of hyperglycaemia with LONS [14, 15, 18].

Our study confirms earlier observations that preterm infants have an increased risk of glucosuria and that glucosuria is negatively associated with gestational age and birth weight [14, 16, 21]. Our study is the first, however, to assess the value of the presence and degree of glucosuria as an early marker in detecting LONS in preterm infants.

### Strengths and weaknesses

The strength of our study is that the data were prospectively collected in a well-defined population of preterm infants suspected of late-onset sepsis. This is important because both clinical symptoms and inflammatory responses may differ between preterm and term infants [24]. Attending physicians and researchers who classified the sepsis episodes were strictly blinded to glucosuria results. This is crucial to prevent incorporation bias and thus overestimation of diagnostic accuracy or associations [25].

We consider it to be an additional strength that we did not only include blood-culture proven sepsis, but also clinical blood-culture negative sepsis, reflecting daily practice in neonatal intensive care wards.

An important weakness of our study was the lack of a strict definition of suspicion of sepsis. We left it up to the attending physician to decide whether the signs and symptoms gave rise to clinical suspicion of a LONS, realizing that this will lead to a certain inter-physician variation. However, the definition of the outcome - confirmed LONS - was clear and decided on by the researchers. Another point of criticism is that we only analyzed the glucosuria results in suspected episodes of LONS and thus did not further analyse the predictive value of glucosuria. This means that we do not know how often preterms experience a change in degree of glucosuria without subsequent clinical suspicion of sepsis. It would be interesting to know whether monitoring of daily glucosuria, is of additional value above monitoring of clinical findings and vital parameters, for early detection of LONS.

## Conclusions

In conclusion, we demonstrated that changes in the degree of glucosuria occur early in the course of LONS in preterm infants, but that this marker is only of marginal diagnostic value.

## Abbreviations

LONS: Late-onset neonatal sepsis
GA: Gestational age
NICU: Neonatal intensive care unit
CRP: C-reactive protein
OR: Odds ratio
CI: Confidence interval
